# Supplementary material for: siRNA-Mediated MELK Knockdown Induces Accelerated Wound Healing with Increased Collagen Deposition
Source: Int J Mol Sci. 2023 Jan 10;24(2):1326. doi: 10.3390/ijms24021326 (PMC9861445; doi:10.3390/ijms24021326)
Supplement: Supplementary file 1 [file ijms-24-01326-s001.zip › ijms-2119979-supplementary.pdf]

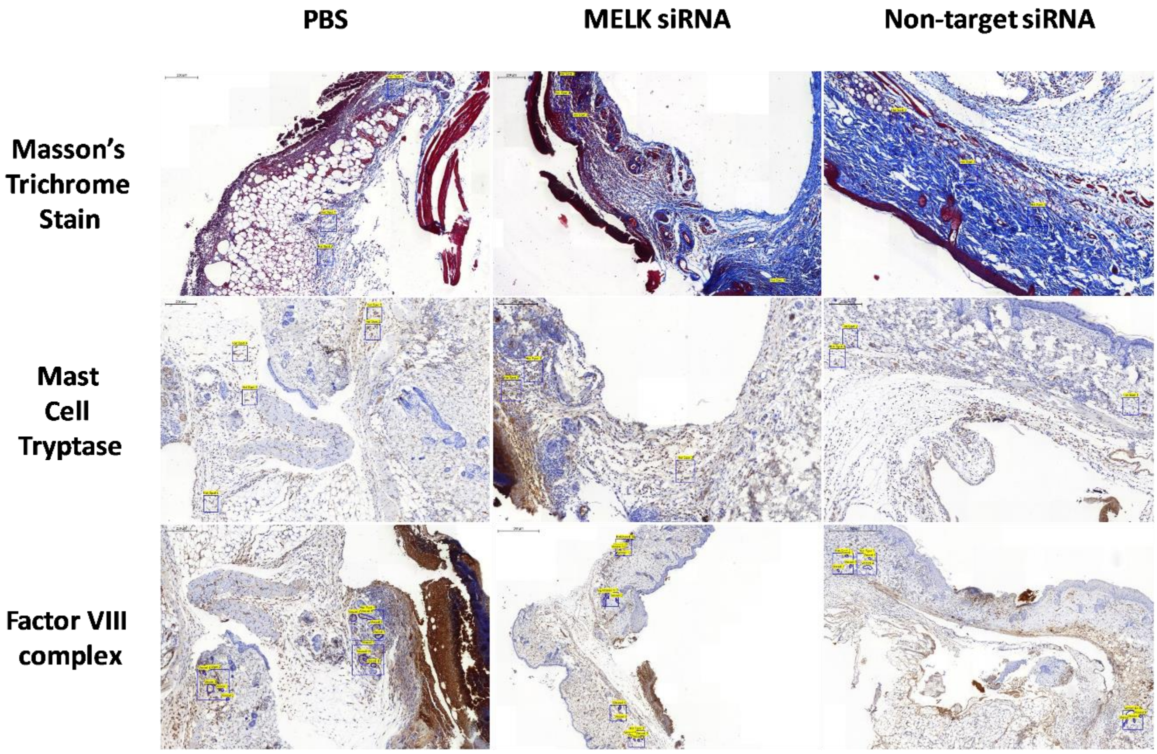

Figure S1. Representative immunohistological staining images. of 3 day wounds. Images were acquired at 400x microscopic magnification using Pannoramic 250 Flash II scanner. Slides were stained with (first line) Masson's Trichrome Stain, (second line) Mast Cell Tryptase antibody, and (third line) von Willebrand Factor/Factor VIII complex antibody.

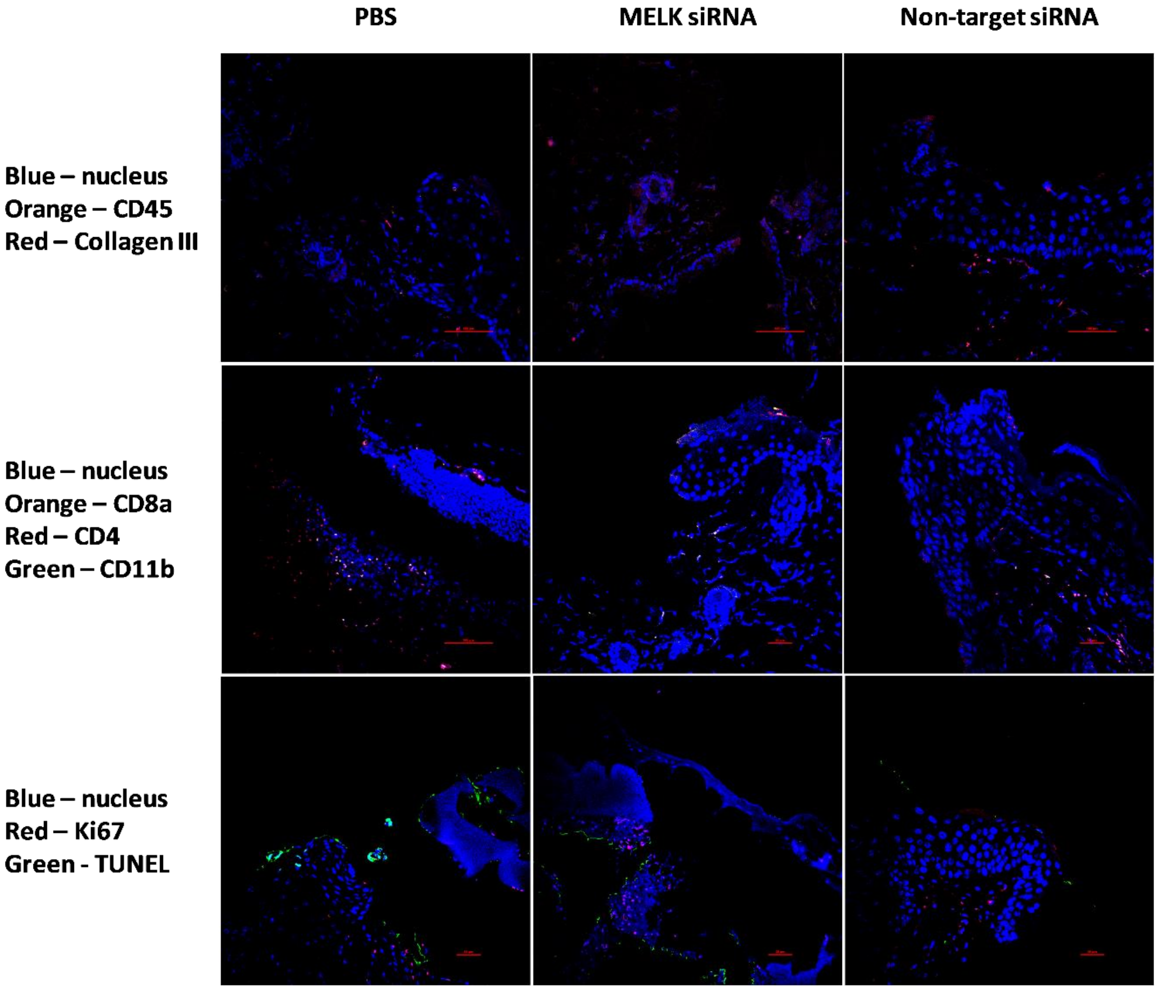

Figure S2. Representative immunofluorescence staining images of 3 day wounds. Images were acquired at 200x microscopic magnification using Nikon A1R confocal microscope. Slides were stained with (first line) CD45 (# 14-0451-82) – orange, collagen III (# PA5-34787) – red, and DAPI – blue; (second line) CD8a (# 50-0081-82) – orange, CD4 (# 41-0042-82) – red, CD11b (# 53-0112-82) – green, and DAPI – blue; (third line) Ki67 (# 41-5698-82) – red, Click-iT Plus TUNEL Assay Kit (# C10617) – green, and DAPI – blue.
